# INNOVATIVE TECHNOLOGIES: Peptide Arrays Break the Species Barrier

**DOI:** 10.1289/ehp.117-a194

**Published:** 2009-05

**Authors:** Tim Lougheed

Phosphorylation, in which phosphate groups attach to proteins or small molecules, is among the cell’s primary means of controlling protein activity. It regulates nearly every aspect of cell life, including cell metabolism, proliferation, viability, differentiation, apoptosis, and transmission of hormone and growth factor signals. Protein kinases are the enzymes that catalyze this myriad of molecular transactions. With more than 500 unique kinases and one-third of mammalian protein expression known to be associated with phosphorylation events, the study of kinase activity via high-throughput analysis represents a high priority for pharmaceutical researchers seeking drug candidates and environmental researchers seeking harmful agents.

Phosphorylation events and characteristics of the kinome (the set of protein kinases in an organism’s genome) are well defined for mice, but mouse models are available only for a subset of human disease states. Other species may offer more accurate models for specific disease or physical conditions in humans, but the phosphorylation profiles of many animals are largely unknown. What is known, however, is the unique composition of the genomes of an ever-expanding number of species. And that turns out to be enough to assemble a peptide array to profile a cell’s kinase activity in a quick and cost-effective fashion, as investigators at the University of Saskatchewan have demonstrated.

“It turns out the protein sequences can be predicted,” explains Scott Napper, a program manager and scientist with the university’s Vaccine and Infectious Disease Organization and International Vaccine Centre (VIDO/InterVac). “You don’t have to rely on existing phosphorylation databases. You can extrapolate from these into other species.”

In a paper published in the 20 January 2009 issue of *Science Signaling*, Napper and his colleagues offer what amounts to a recipe for this sequence-predicting process. In a nutshell, the process relies on the fact that amino acid sequences surrounding phosphorylation sites within specific proteins—along with the biologic function of those phosphorylation events—are often conserved from one species to another.

As a proof-of-principle demonstration focusing on specific proteins previously shown to undergo phosphorylation in human and mouse cells, Napper’s team created a custom array of peptides representing the corresponding proteins in cow cells. Specifically, they identified phosphorylation events relevant to pathways of interest, used publicly available databases to identify human peptide sequences for the phosphorylation sites, searched for bovine peptides that matched the human sequences, and confirmed that the human and bovine peptides were from functionally equivalent proteins. The phosphorylation sites were then arrayed onto slides and exposed to protein kinases from cow cells to profile phosphorylation activity.

Napper describes the approach as remarkably cost effective. An array revealing 300 different phosphorylation events takes about two days and $100 to produce, says Napper. Obtaining these data using a more conventional approach would take much longer, and the price tag could run into tens of thousands of dollars. Moreover, in many cases, species-specific antibodies simply may not be available. “The peptide arrays optimize the amount of data collected yet minimize the amount of laboratory waste generated, compared with traditional approaches,” says Napper. “In addition, we have adjusted the experimental protocol so that radioactivity is no longer required for signal detection.”

The motivation for developing the original peptide array stemmed from Napper’s ongoing work on Johne (“yo-knee”) disease, an intestinal infection that leads to fatal wasting in cattle, sheep, and other ruminants. The new array may provide a quick, practical way to solve the long-standing problem of this disease, Napper says. “By providing insight into the mechanisms of various pathogens of livestock animals, these arrays have the potential to facilitate new treatments that will reduce the prevalence and shedding of these pathogens into the environment,” he says.

Napper adds that the peptide array has made it possible to obtain preliminary insights into mechanisms by which bacteria appear to shut down the host’s immune response. Implications of treating Johne disease could extend beyond veterinary circles: the bacterium responsible for this condition may be associated with Crohn disease in humans, as reported in a review in the January 2008 *Current Opinion in Gastroenterology*. An understanding of the phosphorylation events responsible for Johne disease could point the way to treatments for Crohn disease. Moreover, says B. Alex Merrick, a staff scientist in the NIEHS Laboratory of Respiratory Biology, testing for inflammatory signaling with peptide arrays like this might be useful for screening everything from investigational new drugs to environmental pathogens.

“What is key, and the reason we are excited about it, is the biological information that can come out of it,” says Andrew Potter, director and CEO of VIDO/InterVac. “If we can generate new insights to solve some of the problems we’re working on, that’ll be a huge step forward.”

## Figures and Tables

**Figure:**
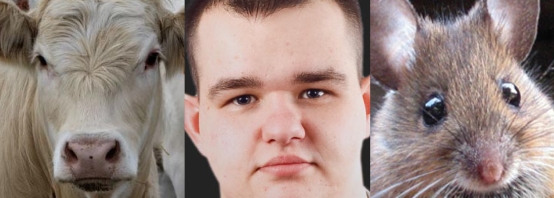
The nuts and bolts of phosphorylation, which regulates nearly every aspect of cell life, are largely conserved across species.

